# Patterns of Transmitted HIV Drug Resistance in Europe Vary by Risk Group

**DOI:** 10.1371/journal.pone.0094495

**Published:** 2014-04-10

**Authors:** Dineke Frentz, David van de Vijver, Ana Abecasis, Jan Albert, Osamah Hamouda, Louise Jørgensen, Claudia Kücherer, Daniel Struck, Jean-Claude Schmit, Jurgen Vercauteren, Birgitta Åsjö, Claudia Balotta, Colm Bergin, Danail Beshkov, Ricardo Camacho, Bonaventura Clotet, Algirdas Griskevicius, Zehava Grossman, Andrzej Horban, Tatjana Kolupajeva, Klaus Korn, Leondios Kostrikis, Kirsi Liitsola Marek Linka, Claus Nielsen, Dan Otelea, Dimitrios Paraskevis, Roger Paredes, Mario Poljak, Elisabeth Puchhammer-Stöckl, Anders Sönnerborg, Danica Stanekova, Maja Stanojevic, Anne-Mieke Vandamme, Charles Boucher, Annemarie Wensing31* on behalf of the SPREAD Programme

**Affiliations:** 1 Department of virology, Erasmus Medical Center, Rotterdam, the Netherlands; 2 Centro de Malária e outras Doenças Tropicais, Instituto de Higiene e Medicina Tropical, Universidade Nova de Lisboa, Lisboa, Portugal; 3 Department of Microbiology, Tumor and Cell Biology, Karolinska Institutet, Stockholm, Sweden; 4 Department of Clinical Microbiology, Karolinska University Hospital, Stockholm, Sweden; 5 Robert Koch Institute, Berlin, Germany; 6 Statens Serum Institute, Copenhagen, Denmark; 7 Laboratory of Retrovirology, CRP-Santé, Luxembourg, Luxembourg; 8 Centre Hospitalier de Luxembourg, Luxembourg; 9 Rega Institute, Katholieke Universiteit Leuven, Leuven, Belgium; 10 Section for Microbiology and Immunology, The Gade Institute, University of Bergen, Bergen, Norway; 11 University of Milan, Milan, Italy; 12 Department of GU Medicine & Infectious Diseases, St James's Hospital, Dublin, Ireland; 13 Department of Virology, National Center of Infectious and Parasitic Diseases, Sofia, Bulgaria; 14 Hospital Egas Moniz, Centro Hospitalar de Lisboa Ocidental, Lisboa, Portugal; 15 irsiCaixa AIDS Research Institute & Lluita contra la SIDA Foundation, Hospital Universitari "Germans Trias i Pujol," Badalona, Spain; 16 National Public Health Surveillance Laboratory, Vilnius, Lithuania; 17 Sheba Medical Center, Tel Hashomer, Israel; 18 Warsaw Medical University and Hospital of Infectious Diseases, Warsaw, Poland; 19 Infectology Center of Latvia, Riga, Latvia; 20 University of Erlangen-Nuremberg, Erlangen, Germany; 21 University of Cyprus, Nicosia, Cyprus; 22 National Institute for Health and Welfare, Helsinki, Finland; 23 National Institute of Public Health, Prague, Czech Republic; 24 Molecular Diagnostics, "Prof Dr Matei Bals" Institute for Infectious Diseases, Bucharest, Romania; 25 Medical School, University of Athens, Athens, Greece; 26 University of Ljubljana, Ljubljana, Slovenia; 27 Medical University Vienna, Vienna, Austria; 28 Divisions of Infectious Diseases and Clinical Virology, Karolinska Institute, Stockholm, Sweden; 29 Slovak Medical University, Bratislava, Slovakia; 30 University of Belgrade School of Medicine, Belgrade, Serbia; 31 Department of Medical Microbiology,University Medical Center Utrecht, Utrecht, the Netherlands; University of Pittsburgh, United States of America

## Abstract

**Background:**

In Europe, a continuous programme (SPREAD) has been in place for ten years to study transmission of drug resistant HIV. We analysed time trends of transmitted drug resistance mutations (TDRM) in relation to the risk behaviour reported.

**Methods:**

HIV-1 patients newly diagnosed in 27 countries from 2002 through 2007 were included. Inclusion was representative for risk group and geographical distribution in the participating countries in Europe. Trends over time were calculated by logistic regression.

**Results:**

From the 4317 patients included, the majority was men-having-sex-with-men -MSM (2084, 48%), followed by heterosexuals (1501, 35%) and injection drug users (IDU) (355, 8%). MSM were more often from Western Europe origin, infected with subtype B virus, and recently infected (<1 year) (p<0.001). The prevalence of TDRM was highest in MSM (prevalence of 11.1%), followed by heterosexuals (6.6%) and IDU (5.1%, p<0.001). TDRM was predominantly ascribed to nucleoside reverse transcriptase inhibitors (NRTI) with a prevalence of 6.6% in MSM, 3.3% in heterosexuals and 2.0% in IDU (p = 0.001). A significant increase in resistance to non- nucleoside reverse transcriptase inhibitors (NNRTIs) and a decrease in resistance to protease inhibitors was observed in MSM (p = 0.008 and p = 0.006, respectively), but not in heterosexual patients (p = 0.68 and p = 0.14, respectively).

**Conclusions:**

MSM showed to have significantly higher TDRM prevalence compared to heterosexuals and IDU. The increasing NNRTI resistance in MSM is likely to negatively influence the therapy response of first-line therapy, as most include NNRTI drugs.

## Introduction

Antiretroviral therapy has strongly reduced morbidity and mortality in HIV infected individuals [1]. This use of antiretroviral medication, however, also led to transmission of drug resistant HIV-1. Transmission of drug resistance has important clinical ramifications as it is associated with an increased probability for virological failure [2]. Importantly, the problem is large, with prevalence ranging between 10 and 15% of antiretroviral naïve patients infected with a virus carrying at least one transmitted drug resistance associated mutation (TDRM) in Europe [3]–[6] and North America [6]–[8].

The prevalence of TDRM is expected to be different among different routes of transmission in Europe. Men having sex with men (MSM) are mostly originating from resource-rich countries where antiretroviral drugs have been available for many years. Until the early 1990s, HIV patients received mono- or dual-therapy with nucleoside reverse transcriptase inhibitors (NRTI). This mono- and dual-therapy led to a rapid selection of drug resistant viruses [9], [10]. In contrast, heterosexually infected patients in Europe are mostly immigrants from Sub-Saharan Africa or individuals from Eastern Europe areas where the use of antiretrovirals in this case in the form of combination antiretroviral therapy has been initiated more recently. These differences in treatment history between the risk groups are reflected in several studies showing a higher likelihood in MSM to be infected with resistant virus compared to other risk groups [3], [11], [12].

While access to antiretrovirals has rapidly been scaled-up during the past decade in Eastern Europe and Africa, TDRM remains limited with a general prevalence of <5% in these areas [13]. However, studies have shown that in some regions in sub-Saharan Africa TDRM is already as high as 11.6% [14]. TDRM in Africa is specifically associated with non-nucleoside reverse transcriptase inhibitors (NNRTIs), which is consistent with the use of single-dose nevirapine to prevent mother-to-child transmission and NNRTI based combinations of antiretroviral therapy (cART) [15].

These global differences in the use of cART may have influenced TDRM in Europe over time. Yet, there are no studies performed which analyse time trends in the various risk groups European-wide. We examined the prevalence of TDRM for the individual drug classes between various HIV risk groups in Europe and to study temporal trends of TDRM in these subgroups in a large European surveillance programme.

## Methods

### Ethics statement

Ethical requirements are fulfilled according to the procedure described in the EC contract. The procedure differs among the 32 countries in the network according to national legislation. Briefly, for each participating hospital or collection center, approval was obtained by the institutional medical ethical review committee. Additionally, a written informed consent was obtained for each patient. In countries where a mandatory surveillance system was already established, legally no informed consent was needed. All surveillance data were made anonymous and coded at national level.

### Study population

Our analyses included data from the SPREAD Programme. The SPREAD programme recruited individuals newly diagnosed with HIV-1 from September 2002 through December 2007 in 27 European countries. For each country a surveillance strategy was defined based on the following criteria: random samples were obtained in those countries in which there was access to more than 80% of all patients newly diagnosed. Alternatively, a pre-defined sampling strategy was used based on the geographical and risk group distribution of newly diagnosed individuals in countries which did not have access to at least 80% of newly diagnosed patients. These approaches pursuit representative sampling of newly diagnosed patients in the participating countries. For more details on the sampling strategy, see the previous reports of the SPREAD Programme [3], [16]. Epidemiological, clinical, and behavioral data were collected using a standardized questionnaire within six months of diagnosis.

A blood sample was taken for genotypic resistance testing within six months after diagnosis. Population-based nucleotide sequencing of the reverse transcriptase and protease genes of the virus was performed at local laboratories by means of commercially available kits or in-house methods. TDRM was defined according to the mutation list published for surveillance of TDRM as recommended by the World Health Organization [17].

Seroconversion was documented in a proportion of the newly diagnosed patients. For 882 patients, a recent infection could be established because a last negative HIV antibody test was available within 3 years before diagnosis. In these patients, the date of infection was estimated as the midpoint between the date of the last negative and first positive test. In addition, for 506 patients primary HIV-1 infection was documented based on the clinical symptoms reported by the treating physician. In these 506 patients, the date of the first positive (and subsequently confirmed) HIV test was used as the estimated date of infection. Since there are limitations to the accuracy of such estimates, the results obtained should be interpreted with care. Patients were defined as recently infected (n = 1236) when the duration of infection was less than 1 year. In our analyses, we use both the total number of recently infected patients and separately the number of recently infected patients where seroconversion was proven by a last negative HIV antibody test.

### Statistical analyses

The HIV-1 subtypes were determined by use of the Rega HIV-1 subtyping tool (version 2.0, available at http://www.bioafrica.net/subtypetool/html/) [18]. The data were analyzed using the statistical software R (version 2.11.1). Prevalence values were calculated with a 95% Wilson score confidence interval (CI) on the basis of a binomial distribution. Categorical data were compared using the chi-square test, Fisher's exact test, or logistic regression techniques. Continuous data were investigated by means of a Mann-Whitney *U*-test or the Kruskal Wallis test.

Trends in the prevalence of TDRM were calculated by multivariate logistic regression in which we included all univariate co-variates with p<0.1.

## Results

### Population characteristics

A total of 4317 newly diagnosed HIV-1 patients were included in the SPREAD programme from September 2002 through December 2007. From these 4317 patients, the majority (2084, 48.3%) were MSM, followed by heterosexuals (1501, 34.8%) and injection drug users (IDU) (355, 8.2%). The baseline characteristics of these three risk groups are summarized in Table 1. MSM were more often recently infected (<1 year) (43.0%) than IDU (23.4%) and heterosexuals (13.5%) (p<0.001). Within the heterosexuals and IDU risk groups, a high ratio of patients had an unknown date of infection (85% and 73%, respectively). The percentage of the subgroup of recently infected with proven seroconversion by last negative HIV test was 30.7% in MSM, 23.4% in IDU, and 8.9% in heterosexuals. As expected from the higher proportion of recent infections, MSM had a higher median CD4 cell count (435, interquartile range (IQR) 259–585 cells/mm^3^) than the corresponding CD4 values found in heterosexuals (median 280, IQR 110–458 cells/mm^3^) and in IDU (median 392, IQR 197–521 cells/mm^3^) (p<0.0001). Furthermore, the proportion originating from Western Europe was highest in MSM (69.9%), followed by IDU (50.4%) and heterosexuals (39.2%) (p<0.0001). In 51.0% of heterosexuals, and 75.8% of IDU, patients were male. As previously reported [19], large differences were seen in subtype distribution (p<0.0001). The most reported HIV subtype in viral isolates from MSM was B (90.4%) whereas in IDU and heterosexuals subtype B was only seen in 61.4% and 33.5%, respectively. In IDU, the most commonly found non-B subtype was subtype A, which was observed in 22.2%. In heterosexuals, both subtype A (18.3%) and subtype C (17.3%) were the most frequently observed non-B subtypes.

**Table 1:** Characteristics of HIV infected individuals.

Characteristics	Categories	MSM (%)	Heterosexuals (%)	IDU (%)	p-value
**HIV patients**		2084	1501	355	
**Origin**	Western Europe	1457 (69.9)	589 (39.2)	179 (50.4)	<0.001
	Eastern Europe & Central Asia	378 (18.1)	253 (16.9)	145 (40.8)	
	Sub-Saharan Africa	10 (0.5)	451 (30.0)	4 (1.1)	
	Other	239 (11.5)	208 (13.9)	27 (7.6)	
**Baseline values**	Plasma HIV-RNA, median (IQR), log copies/ml	4.88 (4.3–5.4)	4.79 (4.2–5.3)	4.76 (4.2–5.3)	<0.001
	CD4 cell count, median (IQR), cells/mm^3^	435 (259–585)	280 (110–458)	392 (197–521)	<0.001
	Age, mean years (IQR)	36.1 (29–41)	37.7 (29–45)	33.2 (26–39)	<0.001
**Gender**	Male	2070 (99.3)	765 (51.0)	269 (75.8)	<0.001
**CDC stage**	A and B	1860 (89.3)	1148 (76.5)	277 (78.0)	<0.001
	C	167 (8.0)	250 (16.7)	37 (10.4)	
**Subtype**	B	1884 (90.4)	503 (33.5)	218 (61.4)	<0.001
	A	86 (4.1)	275 (18.3)	79 (22.2)	
	C	15 (0.7)	259 (17.3)	6 (1.7)	
	02_AG	22 (1.1)	158 (10.5)	5 (1.4)	
	G	11 (0.5)	96 (6.4)	24 (6.8)	
	F	10 (0.5)	54 (3.6)	2 (0.6)	
	others	30 (1.4)	115 (7.7)	16 (4.5)	
	unassigned	26 (1.2)	41 (2.7)	5 (1.4)	
	non-B	174 (8.3)	957 (63.8)	132 (37.2)	
**Duration of infection**	<1 year	897 (43.0)	203 (13.5)	83 (23.4)	<0.001
	1–2 years	108 (5.2)	23 (1.5)	12 (3.4)	
	Unknown duration	1079 (51.8)	1275 (84.9)	260 (73.2)	

**NOTE**. Data are no. (%) of individuals, unless otherwise indicated. Characteristics describe individuals from whom a baseline HIV-1 genotypic analysis was available. CDC, Centers for Disease Control and Prevention; IQR, interquartile ranges; MSM, men who have sex with men; IDU, injection drug users.

### Genotypic resistance analysis

**Figure 1:**
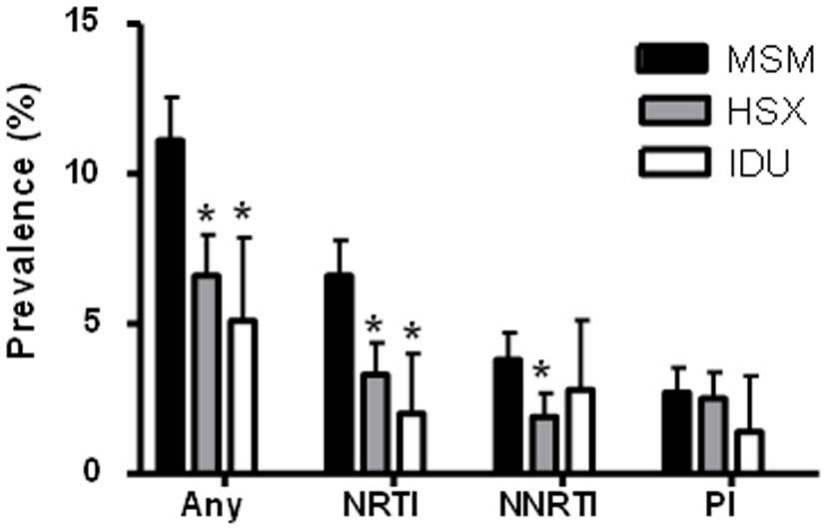
Prevalence of TDRM by drug classes in three risk groups. Prevalences are shown of resistance to at least one of the drug classes (Any), nucleoside reverse transcriptase inhibitor (NRTI), non-nucleoside reverse transcriptase inhibitor (NNRTI) and protease inhibitor (PI) in men who have sex with men (MSM), heterosexuals (HSX), and injection drug users (IDU). * = p<0.001 in comparison with MSM

The prevalence of individuals with at least one TDRM in MSM was 11.1% (95% CI: 9.9–12.6%). This was significantly higher (p <0.001) than in heterosexuals (6.6%; 95% CI: 5.4–8.0%) and IDU (5.1%; 95% CI: 3.2–7.9%) (Figure 1). Similarly, the prevalence of TDRM for nucleoside reverse transcriptase inhibitors (NRTIs), was at least twice as high in MSM (6.6%; 95% CI: 5.6–7.8%) compared to heterosexuals (3.3%; 95% CI: 2.5–4.4%; p<0.001) or IDUs (2.0%; 95% CI: 1.0–4.0%; p = 0.001). Most of the NRTI TDRM were thymidine analogue mutations (TAMs) in MSM (87.7%), in heterosexuals (70.0%) and in IDU (100%).

Also for the non-nucleoside reverse transcriptase inhibitor (NNRTI) TDRM, the prevalence in MSM (3.8%; 95% CI: 3.1–4.7%) was significantly higher compared to heterosexuals (1.9%; 95% CI: 1.3–2.7%; p<0.001) but not to IDU (2.8%; 95% CI: 2.3–1.3%; p = 0.44).

Notably, the prevalence of TDR for NNRTIs was higher than for NRTIs in IDUs, but not statistically significant. The most prevalent NNRTI TDRM was K103N (>57% in all three risk groups).

In the protease inhibitor drug class, no statistically significant differences were seen between the risk groups. This may be due to the low proportions of individuals with protease inhibitor TDRM found in all risk groups; MSM 2.7%, heterosexuals 2.5%; IDU 1.4%). The most prevalent transmitted mutation was the L90M (>24% in all three risk groups).

A large proportion (61%) of the heterosexuals did not originate from Western Europe (60.8%) or North America (0.06%). Of patients not originating from Western Europe or North America, 51.8% were from Sub-Saharan Africa. The prevalence of TDRM in heterosexuals from Western Europe or North America was 7.8% for overall, 4.1% for NRTI, 2.0% for NNRTI and 2.5% for protease inhibitor resistance. These prevalences did not differ significantly from the prevalence of resistance in heterosexuals from non-Western countries (6.0% for overall; p = 0.17, 2.9% for NRTI; p = 0.24, 1.8% for NNRTI; p = 0.85, and 2.5% for protease inhibitor; p = 1).

Interestingly, when individuals from outside Western Europe or North America were excluded from the analyses, the prevalence of TDRM remained significantly different between MSM and heterosexuals for overall TDRM (p = 0.02), NRTI (p = 0.04) and NNRTI (p = 0.02). When we further limited our analysis to just those recently infected from these regions (within 1 year), we found a TDRM prevalence of 13.1% in MSM and 6.8% in heterosexuals (13.3% and 9.7%, respectively, in recently infected proven by a last negative HIV test). In chronically infected patients this prevalence was 9.6% in MSM and 8.0% in heterosexuals (10.2% and 7.8%, respectively, in all patients without a proven seroconversion by a last negative HIV test). The difference in TDRM prevalence could therefore only for a small part be explained by the difference in duration of infection between MSM and heterosexuals.

### Time trends

**Figure 2:**
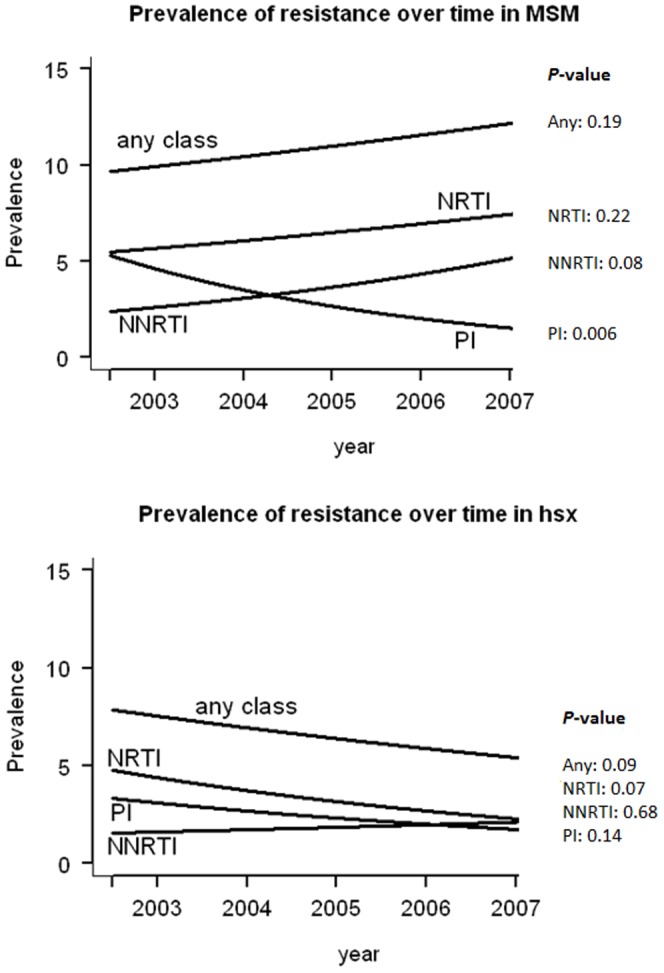
Prevalence of TDRM in patients diagnosed from 2002 through 2007. Prevalence of TDRM is shown for any of the drug classes (any class), nucleoside reverse transcriptase inhibitor (NRTI), non-nucleoside reverse transcriptase inhibitor (NNRTI) and protease inhibitor (PI) in **(A)** Men having sex with men (MSM), and in **(B)** heterosexuals (HSX). The p-values of the time trends are shown on the right side of the graph.

Trends over time were examined in MSM and heterosexuals, but not for IDU as the numbers were too low. The prevalence of TDRM slightly increased - but not statistically significant- over time in the MSM group, with 10.1% in 2003 and 12.5% in 2007 (odds ratio [OR], 1.06 [95% CI, 0.97–1.15]; p = 0.19) (Figure 2A). Conversely, the prevalence declined slightly among the heterosexuals with a prevalence of 8.6% in 2003 and 4.9% in 2007 (OR, 0.89 [95% CI, 0.78–1.02]; p = 0.09) (Figure 2B). The NRTI prevalence followed the same time trend as the overall TDRM prevalence, with an OR of 1.07 (95% CI: 0.96–1.19; p = 0.22) in MSM and 0.84 (95% CI: 0.69–1.01; p = 0.07) in the heterosexual group.

In prevalence of resistance to other drug classes, different trends were observed. Importantly, the NNRTI TDRM prevalence increased three fold from 1.7% in 2003 to 5.0% in 2007 in MSM (OR, 1.21 [95% CI, 1.05–1.39]; p = 0.008). For protease inhibitor TDRM, the prevalence significantly decreased over time from 4.6% in 2003 to 2.0% in 2007 (OR, 0.79 [95% CI, 0.66–0.93]; p = 0.006). This increase of NNRTI TDRM and the decrease of protease inhibitor TDRM was not observed in the heterosexuals (p = 0.68 and p = 0.14, respectively). Adjusting for factors significantly associated with TDRM in the univariate analyses did not change any of the time trend effects that were found.

When splitting up heterosexuals into individuals infected in Western Europe/North America and people infected outside these regions, no significant time trends were found for any of the drug classes in the heterosexuals infected in Western Europe/North America. In the heterosexuals infected in regions outside Western Europe and North America, TDRM decreased significantly (p = 0.03) from 7.8% in 2003 to 3.3% in 2007. This is explained by a significant (p = 0.02) decrease in the NRTI TDRM from 4.3% in 2003 to 1.4% in 2007 (data not shown).

## Discussion

We analysed epidemiological and virological data collected over six years from the European HIV drug resistance surveillance program to gain further insight in the risk factors and associated time trends of transmitted resistance in relation to the HIV risk group. MSM have a significantly higher TDRM prevalence of 11.1% compared to 6.6% in heterosexuals and 5.1% in IDU, with the largest difference was found for NRTI TDRM. In addition, the prevalence of NNRTI TDRM was higher in MSM compared to heterosexuals. The prevalence of protease inhibitor TDRM was low in all risk groups.

Similar observations in TDRM were found in the United Kingdom in 2004 to 2006 where MSM showed a significantly higher prevalence compared to other risk groups [11]. Our results are in contrast with a time trend study performed in Canada, where a significant decrease in TDRM was observed in both MSM and the IDU patients but not in heterosexuals [20]. The differences with our study might be due to the sampling in earlier years (1997–2003), the sampling of only recent infected individuals or the smaller sample size (180) in the Canadian study.

A large proportion of HIV-infected were heterosexuals originating from regions outside Western Europe and North America, mostly from Sub-Saharan Africa. In a large part of these regions, access to antiretroviral drugs has only become available at a large scale during recent years. Although transmitted resistance has been reported, it has not developed to such high levels as seen in resource-rich countries [21], although in Uganda and South-Africa already higher levels of TDRM prevalence are found [14].

Of interest, if we only took into account the TDRM prevalence in heterosexuals originating from Western Europe and North America, TDRM was still lower than the prevalence in MSM originating from the same regions. Therefore, the difference in TDRM prevalence between heterosexuals and MSM could not be fully explained by immigration from HIV infected heterosexuals from countries with a short access to therapy. A possible explanation for the lower TDRM prevalence in heterosexuals from Western Europe and North America is that a large proportion is infected by individuals originating from outside these regions. This is supported by a model of Xiridou et al. [22] which showed that a 53% of new HIV infections in the Netherlands was acquired from African migrants mostly (32%) through sexual transmission in the Netherlands.

Another explanation for the lower TDRM prevalence in heterosexuals could be that heterosexuals enrolled in surveillance programs are more likely to have a longer duration of their HIV infection. We indeed observed high CD4 counts in MSM (435 cells/mm^3^) and very low in heterosexuals patients (280 cells/mm^3^), indicating that heterosexuals were often diagnosed at a late stage of their disease. Resistance mutations that are transmitted may revert to drug sensitive virus over time in plasma because they compromise viral replicative capacity. In that case, the resistant virus variants can no longer be detected by population sequencing used in our study, because this method has limitations in the detection of minor populations [23], [24]. In our study, however, we only found a non-significantly lower TDRM prevalence in heterosexuals from Western Europe/North America who were recently infected (6.8%) compared to chronically infected patients from these regions (8.0%). This discrepancy is probably due to the low numbers of recently infected in heterosexuals from Western Europe/North America. Another explanation for the higher TDRM prevalence in MSM might be that TDRM is spread from untreated patients in clusters of MSM forming a sub-epidemic in these patients [25]–[27]. The high TDRM prevalence seen in this study can indeed be explained by revertants and other mutations, which have been shown to persist over time after transmission and which, therefore, may also persist during onward transmission in clusters [28], [29].

In MSM we observed an increase of NNRTI TDRM and decrease in protease inhibitor TDRM. This can be explained by changes in therapies used over time in the Western world. NNRTI are used in first line therapy in many patients and have a low genetic barrier as development of resistance can occur with just a single mutation [30]. Furthermore the use of non-boosted protease inhibitors has decreased over time and selection of resistance to boosted protease inhibitors is rare [31]–[34].

TDRM in IDU was found to be very low in Europe. An explanation for this is the high proportion of IDU that are originating from East and Central Europe. In many countries from this region, the proportion of HIV-1 patients who receive therapy is low and relatively slow increasing [35]–[37]. And even in countries where the access to antiretroviral therapy is high, IDU have lower rates of access [38]–[40]. The highest proportion of resistance in IDU was seen for the NNRTI drug class. This might also be due to the recent increase in access to antiretroviral therapy in IDU. Therefore, this group did not experience the sub-optimal NRTI therapy in the past, but have experienced the increasing use of NNRTIs in Europe.

In this study, time trends for TDRM prevalence were analyzed for MSM and heterosexuals using data from countries all over Europe. Regions in Europe are dissimilar in HIV and TDRM epidemics. For example, Eastern-, Central-, and Western Europe differ in distribution of transmission groups [41], in (prior) access to antiretrovirals [36] and in the size of their epidemic [41]. Therefore, TDRM differences between the HIV risk groups might also be caused by the patients' region of origin. However, region of origin did not change the time trends, which suggest that the time trend found in MSM and in heterosexuals are not caused by a difference over time in the originating region of patients in Europe.

A potential limitation of our study is the methodology used for categorizing risk group, which was done through patient self-reporting. This could lead to misreporting due to the urge to give socially desirable answers. Discrimination can lead to fear of disclosure of being MSM [42], [43].

Another limitation of this study is that the data were collected until 2007. As a consequence, the results may not be applicable to the current situation in Europe. Although, we did not have access to data from 2007 onwards, several studies from across Europe confirm that after 2007, the prevalence of TDR remains the highest among MSM [12], [44]. We therefore believe that the prevalence of TDR remains the highest among MSM.

A strength of our study is the setting in which the SPREAD programme is performed. The SPREAD programme is a large and sufficiently powered pan- European study that has been running since almost ten years. During this time the programme included patients newly diagnosed with HIV using a predefined strategy that is based on the risk group and geographical distribution of HIV in the participating countries.

In conclusion, TDRM prevalence in MSM is high compared to heterosexuals. A specific concern is the increase in NNRTI resistance which increased three times within the study period of five years. This increase is likely to negatively influence the therapy response of first-line therapy, as most include NNRTI drugs. Therefore, special attention is needed to the further development of the prevalence of NNRTI TDRM in MSM patients.

## References

[1] Palella FJ Jr, Delaney KM, Moorman AC, Loveless MO, Fuhrer J, et al (1998) Declining morbidity and mortality among patients with advanced human immunodeficiency virus infection. HIV Outpatient Study Investigators. N Engl J Med 338: 853–860. 9516219 10.1056/NEJM199803263381301

[2] Wittkop L, Gunthard HF, de Wolf F, Dunn D, Cozzi-Lepri A, et al. (2011) Effect of transmitted drug resistance on virological and immunological response to initial combination antiretroviral therapy for HIV (EuroCoord-CHAIN joint project): a European multicohort study. Lancet Infect Dis. 10.1016/S1473-3099(11)70032-9 21354861

[3] Vercauteren J, Wensing AM, van de Vijver DA, Albert J, Balotta C, et al (2009) Transmission of drug-resistant HIV-1 is stabilizing in Europe. J Infect Dis 200: 1503–1508. 19835478 10.1086/644505

[4] Bannister WP, Cozzi-Lepri A, Clotet B, Mocroft A, Kjaer J, et al (2008) Transmitted drug resistant HIV-1 and association with virologic and CD4 cell count response to combination antiretroviral therapy in the EuroSIDA Study. J Acquir Immune Defic Syndr 48: 324–333. 18545152 10.1097/QAI.0b013e31817ae5c0

[5] Yerly S, von WV, Ledergerber B, Boni J, Schupbach J, et al (2007) Transmission of HIV-1 drug resistance in Switzerland: a 10-year molecular epidemiology survey. AIDS 21: 2223–2229. 18090050 10.1097/QAD.0b013e3282f0b685

[6] Frentz D, Boucher CA, van de Vijver DA (2012) Temporal Changes in the Epidemiology of Transmission of Drug-Resistant HIV-1 across the World. AIDS Rev 14: 17–27. 22297501

[7] Wheeler WH, Ziebell RA, Zabina H, Pieniazek D, Prejean J, et al (2010) Prevalence of transmitted drug resistance associated mutations and HIV-1 subtypes in new HIV-1 diagnoses, U.S.-2006. AIDS 24: 1203–1212. 20395786 10.1097/QAD.0b013e3283388742

[8] Hurt CB, McCoy SI, Kuruc J, Nelson JA, Kerkau M, et al (2009) Transmitted antiretroviral drug resistance among acute and recent HIV infections in North Carolina from 1998 to 2007. Antivir Ther 14: 673–678. 19704170 PMC2860724

[9] Schuurman R, Nijhuis M, van Leeuwen R, Schipper P, de Jong D, et al (1995) Rapid changes in human immunodeficiency virus type 1 RNA load and appearance of drug-resistant virus populations in persons treated with lamivudine (3TC). J Infect Dis 171: 1411–1419. 7539472 10.1093/infdis/171.6.1411

[10] Boucher CA, O'Sullivan E, Mulder JW, Ramautarsing C, Kellam P, et al (1992) Ordered appearance of zidovudine resistance mutations during treatment of 18 human immunodeficiency virus-positive subjects. J Infect Dis 165: 105–110. 1370174 10.1093/infdis/165.1.105

[11] Booth CL, Garcia-Diaz AM, Youle MS, Johnson MA, Phillips A, et al (2007) Prevalence and predictors of antiretroviral drug resistance in newly diagnosed HIV-1 infection. J Antimicrob Chemother 59: 517–524. 17213262 10.1093/jac/dkl501

[12] Karlsson A, Bjorkman P, Bratt G, Ekvall H, Gisslen M, et al (2012) Low prevalence of transmitted drug resistance in patients newly diagnosed with HIV-1 infection in Sweden 2003–2010. PLoS One 7: e33484. 22448246 10.1371/journal.pone.0033484 PMC3308981

[13] World Health Organization (2011) HIV Drug resistance fact sheet.

[14] Hamers RL, Wallis CL, Kityo C, Siwale M, Mandaliya K, et al (2011) HIV-1 drug resistance in antiretroviral-naive individuals in sub-Saharan Africa after rollout of antiretroviral therapy: a multicentre observational study. Lancet Infect Dis 11: 750–759. 21802367 10.1016/S1473-3099(11)70149-9

[15] Arrive E, Newell ML, Ekouevi DK, Chaix ML, Thiebaut R, et al (2007) Prevalence of resistance to nevirapine in mothers and children after single-dose exposure to prevent vertical transmission of HIV-1: a meta-analysis. Int J Epidemiol 36: 1009–1021. 17533166 10.1093/ije/dym104

[16] SPREAD Programme (2008) Transmission of drug-resistant HIV-1 in Europe remains limited to single classes. AIDS 22: 625–635. 18317004 10.1097/QAD.0b013e3282f5e062

[17] Bennett DE, Camacho RJ, Otelea D, Kuritzkes DR, Fleury H, et al (2009) Drug resistance mutations for surveillance of transmitted HIV-1 drug-resistance: 2009 update. PLoS One 4: e4724. 19266092 10.1371/journal.pone.0004724 PMC2648874

[18] de Oliveira T, Deforche K, Cassol S, Salminen M, Paraskevis D, et al (2005) An automated genotyping system for analysis of HIV-1 and other microbial sequences. Bioinformatics 21: 3797–3800. 16076886 10.1093/bioinformatics/bti607

[19] Abecasis AB, Wensing AM, Paraskevis D, Vercauteren J, Theys K, et al (2013) HIV-1 subtype distribution and its demographic determinants in newly diagnosed patients in Europe suggest highly compartmentalized epidemics. Retrovirology 10: 7. 23317093 10.1186/1742-4690-10-7 PMC3564855

[20] Routy JP, Machouf N, Edwardes MD, Brenner BG, Thomas R, et al (2004) Factors associated with a decrease in the prevalence of drug resistance in newly HIV-1 infected individuals in Montreal. AIDS 18: 2305–2312. 15577543 10.1097/00002030-200411190-00011

[21] UNAIDS (2002) Report on the global HIV/AIDS epidemic, July 2002.

[22] Xiridou M, van Veen M, Coutinho R, Prins M (2010) Can migrants from high-endemic countries cause new HIV outbreaks among heterosexuals in low-endemic countries? AIDS 24: 2081–2088. 20671545 10.1097/QAD.0b013e32833a6071

[23] Metzner KJ, Rauch P, Walter H, Boesecke C, Zollner B, et al (2005) Detection of minor populations of drug-resistant HIV-1 in acute seroconverters. AIDS 19: 1819–1825. 16227789 10.1097/01.aids.0000189878.97480.ed

[24] Schuurman R, Brambilla D, de Groot T, Huang D, Land S, et al (2002) Underestimation of HIV type 1 drug resistance mutations: results from the ENVA-2 genotyping proficiency program. AIDS Res Hum Retroviruses 18: 243–248. 11860670 10.1089/088922202753472801

[25] Brenner BG, Roger M, Routy JP, Moisi D, Ntemgwa M, et al (2007) High rates of forward transmission events after acute/early HIV-1 infection. J Infect Dis 195: 951–959. 17330784 10.1086/512088

[26] Bezemer D, van Sighem A, Lukashov VV, van der Hoek L, Back N, et al (2010) Transmission networks of HIV-1 among men having sex with men in the Netherlands. AIDS 24: 271–282. 20010072 10.1097/QAD.0b013e328333ddee

[27] Yerly S, Vora S, Rizzardi P, Chave JP, Vernazza PL, et al (2001) Acute HIV infection: impact on the spread of HIV and transmission of drug resistance. AIDS 15: 2287–2292. 11698702 10.1097/00002030-200111230-00010

[28] Pingen M, Nijhuis M, de Bruijn JA, Boucher CA, Wensing AM (2011) Evolutionary pathways of transmitted drug-resistant HIV-1. J Antimicrob Chemother. 10.1093/jac/dkr157 21502281

[29] Jain V, Sucupira MC, Bacchetti P, Hartogensis W, Diaz RS, et al (2011) Differential persistence of transmitted HIV-1 drug resistance mutation classes. J Infect Dis 203: 1174–1181. 21451005 10.1093/infdis/jiq167 PMC3107558

[30] Luber AD (2005) Genetic barriers to resistance and impact on clinical response. MedGenMed 7: 69. PMC1681671 16369295

[31] Bartlett JA, Buda JJ, von Scheele B, Mauskopf JA, Davis EA, et al (2006) Minimizing resistance consequences after virologic failure on initial combination therapy: a systematic overview. J Acquir Immune Defic Syndr 41: 323–331. 16540933 10.1097/01.qai.0000197070.69859.f3

[32] Gupta R, Hill A, Sawyer AW, Pillay D (2008) Emergence of drug resistance in HIV type 1-infected patients after receipt of first-line highly active antiretroviral therapy: a systematic review of clinical trials. Clin Infect Dis 47: 712–722. 18662137 10.1086/590943

[33] Lima VD, Gill VS, Yip B, Hogg RS, Montaner JS, et al (2008) Increased resilience to the development of drug resistance with modern boosted protease inhibitor-based highly active antiretroviral therapy. J Infect Dis 198: 51–58. 18498238 10.1086/588675

[34] Mills AM, Nelson M, Jayaweera D, Ruxrungtham K, Cassetti I, et al (2009) Once-daily darunavir/ritonavir vs. lopinavir/ritonavir in treatment-naive, HIV-1-infected patients: 96-week analysis. Aids 23: 1679–1688. 19487905 10.1097/QAD.0b013e32832d7350

[35] Morrison SD, Banushi VH, Sarnquist C, Gashi VH, Osterberg L, et al (2011) Barriers to care and current medical and social needs of HIV-positive patients in Albania. Cent Eur J Public Health 19: 91–97. 21739899 10.21101/cejph.a3644

[36] Bollerup AR, Donoghoe MC, Lazarus JV, Nielsen S, Matic S (2008) Access to highly active antiretroviral therapy (HAART) in the WHO European Region 2003–2005. Scand J Public Health 36: 183–189. 18519283 10.1177/1403494807085191

[37] Donoghoe MC, Bollerup AR, Lazarus JV, Nielsen S, Matic S (2007) Access to highly active antiretroviral therapy (HAART) for injecting drug users in the WHO European Region 2002-2004. Int J Drug Policy 18: 271–280. 17689375 10.1016/j.drugpo.2007.02.010

[38] Celentano DD, Galai N, Sethi AK, Shah NG, Strathdee SA, et al (2001) Time to initiating highly active antiretroviral therapy among HIV-infected injection drug users. AIDS 15: 1707–1715. 11546947 10.1097/00002030-200109070-00015

[39] van Asten LC, Boufassa F, Schiffer V, Brettle RP, Robertson JR, et al (2003) Limited effect of highly active antiretroviral therapy among HIV-positive injecting drug users on the population level. Eur J Public Health 13: 347–349. 14703322 10.1093/eurpub/13.4.347

[40] Wood E, Montaner JS, Yip B, Tyndall MW, Schechter MT, et al (2003) Adherence and plasma HIV RNA responses to highly active antiretroviral therapy among HIV-1 infected injection drug users. CMAJ 169: 656–661. 14517122 PMC202281

[41] European Centre for Disease Prevention and Control, WHO Regional Office for Europe HIV/AIDS surveillance in Europe (2007) Stockholm, European Centre for Disease Prevention and Control, 2008.

[42] Gorbach PM, Galea JT, Amani B, Shin A, Celum C, et al (2004) Don't ask, don't tell: patterns of HIV disclosure among HIV positive men who have sex with men with recent STI practising high risk behaviour in Los Angeles and Seattle. Sex Transm Infect 80: 512–517. 15572626 10.1136/sti.2004.010918 PMC1744943

[43] Shoptaw S, Weiss RE, Munjas B, Hucks-Ortiz C, Young SD, et al (2009) Homonegativity, substance use, sexual risk behaviors, and HIV status in poor and ethnic men who have sex with men in Los Angeles. J Urban Health 86 Suppl 177–92. 19526346 10.1007/s11524-009-9372-5 PMC2705491

[44] Yebra G, Delgado R, Pulido F, Rubio R, Galan JC, et al. (2013) Different trends of transmitted HIV-1 drug resistance in Madrid, Spain, among risk groups in the last decade. Arch Virol. 10.1007/s00705-013-1933-y 24297490

